# Public health challenges and opportunities after COVID-19

**DOI:** 10.2471/BLT.20.267757

**Published:** 2021-04-29

**Authors:** Pier Luigi Sacco, Manlio De Domenico

**Affiliations:** a Department of Humanities, IULM University, Via Carlo Bo, 1, 20143 Milan, Italy.; b CoMuNe Lab, Fondazione Bruno Kessler, Povo, Italy.

## Abstract

With hindsight, the main weakness behind the ineffective response to the coronavirus disease 2019 (COVID-19) pandemic in some countries has been the failure to understand, and take account of, the multilayered systemic interdependencies that spread the effects of the pandemic across social, technological, economic and health-care dimensions. For example, to respond to the COVID-19 pandemic, all people were required to rapidly adjust to social distancing and travel restrictions. Such a complex behavioural response entails adaptation to achieve a full recovery from the systemic shock. To capitalize on the positive effects of disruption to the status quo, much more complex socioeconomic modelling needs to be considered when designing and evaluating possible public health interventions that have major behavioural implications. We provide a simple example of how this reasoning may highlight generally unacknowledged connections and interdependencies and guide the construction of scenarios that can inform policy decisions to enhance the resilience of society and tackle existing societal challenges.

## Résumé

Avec le recul, le principal motif d'inefficacité dans la lutte contre la pandémie de maladie à coronavirus 2019 (COVID-19) dans certains pays trouve son origine dans l'incapacité à comprendre les interdépendances systémiques à de multiples niveaux et à en tenir compte. Ces dernières répercutent les effets de la pandémie sur plusieurs dimensions: sociale, technologique, économique et sanitaire. Pour tenter de contenir la pandémie de COVID-19, la population a notamment été contrainte de se conformer rapidement aux mesures de distanciation physique et aux restrictions de voyage. Un changement de comportement aussi abrupt requiert un temps d'adaptation afin de se remettre totalement d'un tel choc structurel. Si l'on souhaite profiter de l'impact positif qu'exerce ce bouleversement de situation, des modèles socio-économiques bien plus complexes doivent être envisagés au moment de concevoir et d'évaluer les interventions de santé publique potentielles ayant des conséquences majeures sur le comportement. Dans le présent document, nous citons un exemple simple qui montre comment ce raisonnement pourrait mettre en lumière des connexions et interdépendances souvent méconnues, mais aussi guider l'élaboration de scénarios qui serviront à étayer les décisions politiques, accroître la résilience de la société et aborder les enjeux sociétaux actuels.

## Resumen

En retrospectiva, el principal punto débil de la ineficacia de la respuesta a la pandemia de la enfermedad por coronavirus 2019 (COVID-19) en algunos países ha sido la incapacidad de comprender y tener en cuenta las interdependencias sistémicas de varios niveles que difundieron los efectos de la pandemia en las dimensiones social, tecnológica, económica y sanitaria. Por ejemplo, para responder a la pandemia de la COVID-19, todas las personas tuvieron que adaptarse rápidamente al distanciamiento social y a las restricciones de movilidad. Una respuesta conductual tan compleja conlleva la adaptación para lograr una recuperación total del choque sistémico. Para aprovechar los efectos positivos de la alteración del *statu quo*, es necesario tener en cuenta una modelización socioeconómica mucho más compleja a la hora de diseñar y evaluar posibles intervenciones de salud pública que tengan importantes implicaciones conductuales. Aportamos un ejemplo sencillo de cómo este razonamiento puede poner de manifiesto conexiones e interdependencias generalmente no reconocidas y guiar la construcción de escenarios que puedan informar las decisiones políticas para mejorar la resiliencia de la sociedad y abordar los retos sociales existentes.

## ملخص

هناك إدراك متأخر بأن نقطة الضعف الرئيسية التي أدت إلى عدم فعالية الاستجابة لجائحة مرض فيروس كورونا في عام 2019 (كوفيد 19) في بعض البلدان هي عدم فهم الاعتمادات المنهجية المتبادلة متعددة الطبقات ووضعها في الحسبان، وهي التي تؤدي إلى نشر آثار الجائحة عبر الأبعاد الاجتماعية والتقنية والاقتصادية والبُعد الخاص بالرعاية الصحية. للاستجابة مثلاً لجائحة كوفيد 19، كان مطلوبًا من كل الناس أن يتكيفوا سريعًا مع قيود التباعد الاجتماعي والقيود على السفر. إن مثل هذه الاستجابة السلوكية المعقدة تتطلب التكيف لتحقيق التعافي الكامل من الصدمة النظامية. للاعتماد على الآثار الإيجابية لتوقف الوضع القائم، مطلوب التفكير في وضع نماذج اجتماعية اقتصادية أكثر تعقيدًا بكثير عند تصميم وتقييم التدخلات المحتملة الخاصة بالصحة العامة والتي لها تبعات سلوكية كبيرة. نقدم مثالاً بسيطًا على أن طريقة التفكير هذه قد تلقي الضوء على علاقات واعتمادات متبادلة غير معروفة عمومًا وتقدم إرشادات لوضع سيناريوهات يمكن الاعتماد عليها في القرارات المتعلقة بالسياسة لتحسين مرونة المجتمع والتعامل مع التحديات المجتمعية القائمة.

## 摘要

事后看来，在 2019 冠状病毒病（新型冠状病毒肺炎）疫情出现时，某些国家/地区的应对方案效果不佳，其主要原因在于未能理解和考虑到将疫情影响扩散到社会、技术、经济和医疗层面的多层系统相互依赖性。例如，为了应对新型冠状病毒肺炎疫情，要求所有人立即保持社交距离并限制出行。这种复杂的行为反应需要时间适应，才能保证整个系统从冲击中完全缓过来。为了充分利用打破现状的积极影响，在设计和评估具有重大行为影响的潜在公共卫生干预措施时，需要考虑复杂得多的社会经济模型。我们提供了一个简单的例子，来说明这种论证如何突出通常未确认的联系和相互依赖关系，并指导构建能够传达政策决定，以增强社会适应力并应对现有社会挑战的场景。

## Резюме

Оглядываясь назад, можно отметить, что реакция на пандемию коронавирусной инфекции 2019 года (COVID-19) в некоторых странах оказалась неэффективной, и основной причиной этого стала неспособность понять и принять во внимание многослойные системные взаимозависимости, из-за которых последствия пандемии затронули одновременно социальные, технологические, экономические и медицинские аспекты. Например, для реагирования на пандемию COVID-19 все люди должны были быстро адаптироваться к социальному дистанцированию и ограничениям на поездки. Такая сложная поведенческая реакция влечет за собой адаптацию для полного восстановления после системного шока. Чтобы извлечь выгоду из положительных последствий нарушения статус-кво, при разработке и оценке возможных мероприятий в области общественного здравоохранения, имеющих серьезные поведенческие последствия, необходимо учитывать гораздо более сложное социально-экономическое моделирование. Авторами приведен простой пример того, как это рассуждение может выявить в целом непризнанные связи и взаимозависимости и повлиять на создание сценариев, которые могут служить основой для принятия политических решений с целью повышения устойчивости общества к сложным ситуациям и увеличения возможностей решения существующих социальных проблем.

## Unexpected shocks

In October 1973, as a result of the sudden embargo called by Arab Members of the Organization of the Petroleum Exporting Countries in retaliation against countries supporting Israel in the Yom Kippur War, oil prices increased greatly in a few months, leading to an overall fourfold increase at the end of the embargo 6 months later. The speed and size of the change took the global economy by surprise and forced the public to face unprecedented social conditions such as empty city streets because of bans on using cars. Even if not apparent at the time, this event was a turning point which had not only short-term effects, but profound long-term ones,^1^ such as contributing to the emergence of the green economy^2^ and to the increasing economic and political relevance of concerns about environmental sustainability.^3^^,^^4^ An unintended consequence of the event was that it became a sort of natural experiment because the public worldwide had a direct experience of what a less fossil-fuel intensive economy and society would look like – something that would have been impossible within any business-as-usual policy agenda, however ambitious or disruptive.^5^

The current coronavirus disease 2019 (COVID-19) pandemic can, to a large extent, be seen as a new example of a large-scale unexpected shock, which is likely to have many complex and long-term consequences on several areas of society and the global economy.^6^^,^^7^ Even more than the car-free weekends of the 1970s, we have faced an unprecedented situation in this pandemic with entire countries having prolonged restrictions, such as curfews, school closures and gathering and travel restrictions. Only a couple of years ago, this kind of event would have been confined to the realms of science fiction. And yet, such conditions have now become the status quo and the starting point from which we have to design new strategies and policies for development. The pandemic crisis can therefore be seen as an important opportunity for large-scale, deep-seated structural change, far beyond, in scale and scope, what could have reasonably been possible through conventional stakeholder negotiations on typical policy issues. Moreover, the potential level of structural change that could result from the pandemic is far more diverse and multifaceted than the greening of the economy as a result of the 1970s energy shock because the extent and complexity of current technological development exceeds that of half a century ago.^8^

However, the very reason why this shock provides new opportunities for societal development is also the reason for its serious social and economic consequences: the fact that it was unthinkable by pre-pandemic standards. Although many experts in the past few decades repeatedly warned that the occurrence of a global pandemic was a near certainty,^9^^,^^10^ these calls were not taken seriously enough. Furthermore, with a few exceptions, the COVID-19 pandemic found many public health and social systems unprepared, including the systems of most socioeconomically developed countries such as the United States of America (USA).^11^ People seem unable to consider major catastrophic changes as a real possibility before they happen, and to take into account their knock-on effects.^12^ With hindsight, the main weakness behind the ineffective response to the pandemic has been the failure to understand, and take account of, the multilayered systemic interdependencies that spread the effects of the pandemic across social, technological, economic and health-care dimensions. In other words, policy failures in the context of the current pandemic can be traced to the inability to think in terms of the emergent behavioural responses that are typical of all kinds of complex adaptive systems.^13^ This inability is a particularly serious flaw in a world built around globalization which interlinks global and local dimensions,^14^ whose effects simultaneously appear over many different (and previously considered largely distinct) layers of human activity.

## Unintended consequences

In the case of the COVID-19 crisis, acknowledging how the epidemic dimension coevolved with an infodemic dimension, that is, an excess of not necessarily reliable information, is easy.^15^ These dimensions created complex feedback loops between the dynamics of infection on social behaviour and the dynamics of media content production, dissemination and news consumption. Exposure to a large amount of information, often contradictory, about the pandemic and its many effects on almost all aspects of human existence has deeply influenced individual and social behaviours and, through them, the economic and financial systems, education, the retail industry, logistics and entertainment, and even religion, to mention a few obvious examples. Travel restrictions are forcing work and education to be restructured and decentralized through ad hoc digital platforms, while global travel bans are forcing business and science to move from physical to digital meetings. This situation has led to a rethinking of communication and organizational methods, and probably business models themselves, while accelerating the development of digital technologies that will remodel economic, learning, professional and social systems and environments.

However, we argue whether such structural changes will facilitate the achievement of environmental and social sustainability goals or make their attainment harder, with consequent effects on the related socioeconomic inequalities. Possible advantages of such changes might include an improvement in the work–life balance from new mixed or integral forms of teleworking, which, by reducing work-related movement, might reduce the human impact on climate, thus saving human lives and contributing to lower the risk of future pandemics.^16^ For instance, in China^17^ and northern Italy,^18^ the extensive restrictions which saw a sudden stop to most industrial activity and private motorized transportation brought about an immediate improvement in air quality. The pandemic has also had a noticeable effect on sociopolitical trends. In Italy again, the crisis abruptly disrupted the previous populist, xenophobic orientation of the public discourse, making space for a new dialogue that has shifted to a recovered sense of human solidarity and cohesion.^19^ Other observed effects concern the rebuilding of national pride on the basis of cultural excellence, generosity and the selfless dedication of health professionals,^20^ as an example of the deep roots of Italy’s social and cultural values. Furthermore, a regained sense of respect for the authority of experts, not only in the health professions, has been observed. At the same time, fringe but very vocal social circles such as groups opposed to vaccination and more generally believers in conspiracy theories have lost momentum in the broader public opinion.^21^ However, not all changes have been for the good. Some other countries have witnessed a substantial escalation of conspiratorial thinking^22^ and a strong politicization of the pandemic crisis, which have greatly affected viral transmission and the consequent death rate.^23^

Moreover, any reported positive effects, however encouraging, only highlight specific aspects of the impact of the crisis. Other, concurrent aspects might have serious negative effects, to the point that they more than counteract desirable changes. These effects include for example increasing social isolation, mental health illness, redundancies, financial difficulties and permanent closure of many businesses. The people more likely to be affected by such adverse outcomes are the ones who were already experiencing social, financial and educational deprivation before the pandemic crisis struck. Furthermore, such negative effects are likely to have more impact the less effective a country’s response has been overall. Therefore, we need a systemic approach in both assessing the consequences of the pandemic crisis and in designing adequate response policies, while taking into account social sustainability goals. A good illustration of this point comes from considering the social distancing measures that are necessary to mitigate the impact of the pandemic and to limit infections to levels that the health system has the capacity to manage. Claiming that social distancing is necessary and even inevitable does not imply that it is also socially sustainable. In fact, the social sustainability of social distancing ultimately depends on seemingly unrelated cultural variables, such as widespread perceptions of economic fairness and social privilege. As social distancing prevents many people from earning an income and confines them in their homes, factors such as the availability of savings, quality of living space and family relations inevitably become serious problems, not only politically, but also in terms of effectiveness of public health measures.^24^ If people in disadvantaged positions refuse to comply with social distancing instructions because they perceive them as an unfair toll on their social condition, the effects of an objectively useful and important public health measure could be partly or totally jeopardized.

Despite the wide differences in socioeconomic and cultural characteristics, and the tone and topics of the public discourse across countries, such major crises will probably have a considerable impact on sociocultural orientations and the evolution of the public discourse itself. On the other hand, the late and often contradictory and ill-organized reaction to the crisis by most governments illustrates how many political decision-makers are unprepared to react effectively and promptly to shocks characterized by the functional interdependencies of the social, economic and public health systems. To capitalize on the positive effects of disruption to the status quo, much more complex socioeconomic modelling needs to be considered when designing and evaluating possible public health interventions which have major behavioural implications.

## Post-pandemic scenarios

The speed and size of change caused by large systemic shocks such as the COVID-19 pandemic require a clearly different policy approach to complexity. How can we anticipate and, to some extent, drive such changes if we have no pre-existing experience of the new situation? To what extent are existing theories able to guide our understanding? A suitably designed system-thinking that is able to deal with uncertainty about both the obvious and latent interdependencies of our society might be of help here, if it enables us to analyse and classify the entanglement of its subsystems or layers,^25^^,^^26^ and to predict their evolution. This task is so challenging that it should only be tackled through computational approaches and not top-down reasoning. What could therefore be seen as an exceptional approach to science to address major, unexpected changes would thus become the so-called new normal, namely, the most appropriate way to do science in an era of hyper-connectedness.

**Fig. 1:**
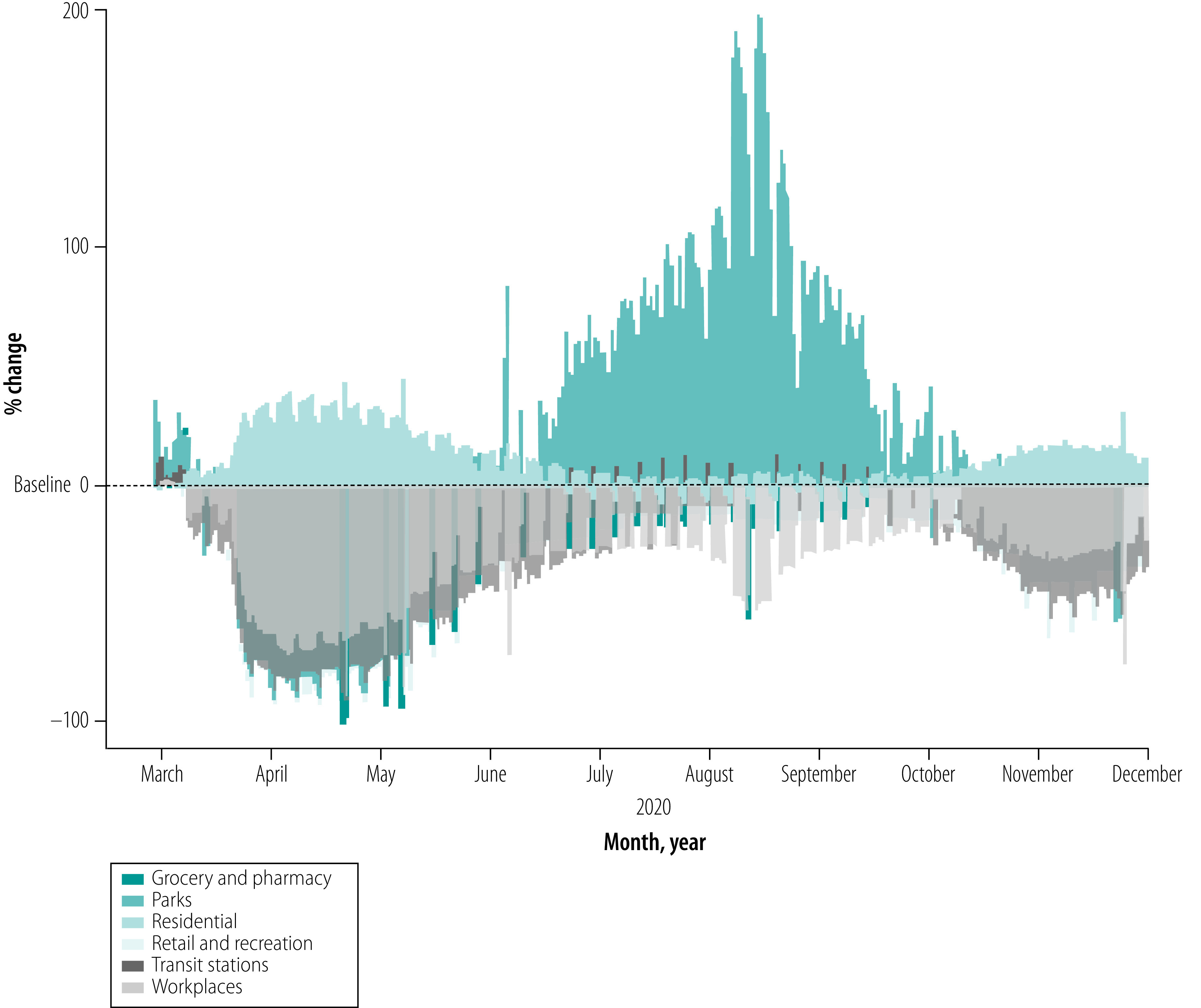
Effects of travel restrictions and social distancing on human movement during the COVID-19 pandemic, Italy, March to early December 2020

**Fig. 2:**
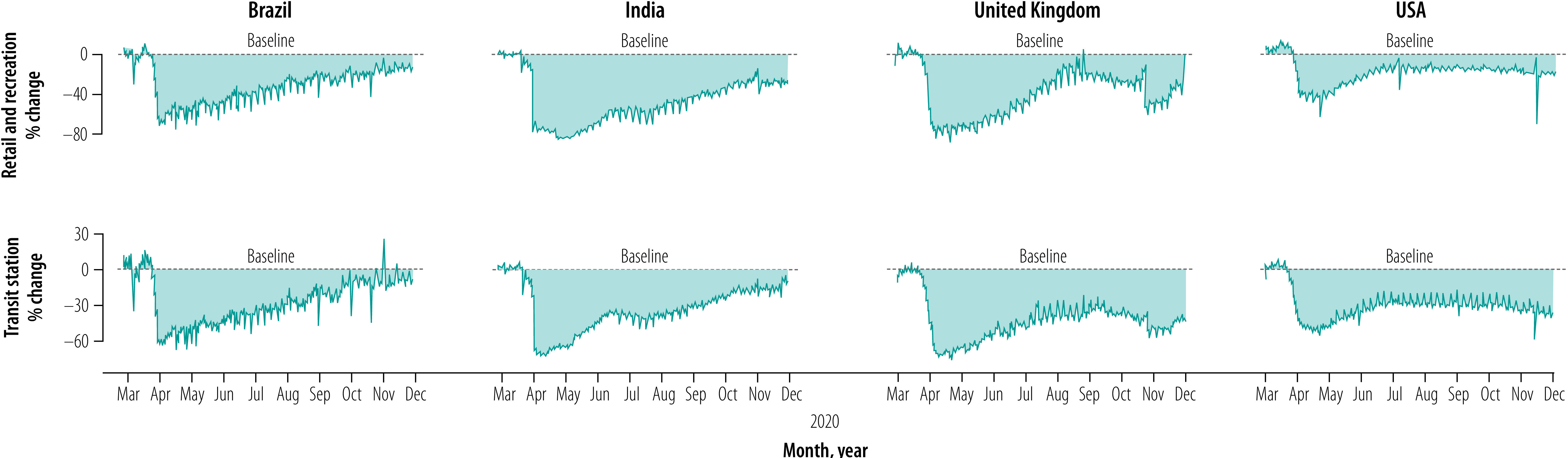
Effects of travel restrictions and social distancing on human movement during the COVID-19 pandemic in selected countries, March to early December 2020

Developing a full model of the inter-relatedness of the various kinds of systems in a pandemic crisis is beyond the scope of this article. However, we provide a simple example of how this line of thinking may highlight generally unacknowledged connections and guide the construction of possible scenarios that can inform policy evaluations and decisions. Based on Google mobility data from March to early December 2020, we report the impact of pandemic-related movement restrictions and social distancing on various dimensions of socioeconomic activity in Italy and a few countries representative of different continents (Fig. 1 and Fig. 2). We see a substantial shift in mobility behaviour compared with the pre-pandemic baseline, with a collapse of work-, retail- and recreation-related movement in favour of residential- and nature-related movement. Moreover, in various countries, such changes in movement seem to have stabilized considerably under baseline levels, especially in socioeconomically advanced countries such as the United Kingdom of Great Britain and Northern Ireland and the USA (Fig. 2). On the other hand, in less socioeconomically developed countries, such as Brazil and India, where most people have much smaller savings buffers and less possibility to make use of technology to reorganize their business in the context of low levels of movement of people, the level of movement tends to return to the baseline even though the pandemic crisis is far from over. In fact, movement is increasing, as in the case of Brazil and India (Fig. 2).^28^^,^^29^

COVID-19: coronavirus disease 2019.

Note: We used Google to measure the effects of travel restrictions and social distancing on human movement through the location history of its users.^27^

COVID-19: coronavirus disease 2019.

Note: We used Google to measure the effects of travel restrictions and social distancing on human movement through the location history of its users.^27^

We built this example using a single data source. However, it shows how, by only considering movement, much can be understood about the complex dynamic of behavioural change that is triggered by a large, unexpected crisis, the persistence of its effects and the possible important determinants.

**Fig. 3:**
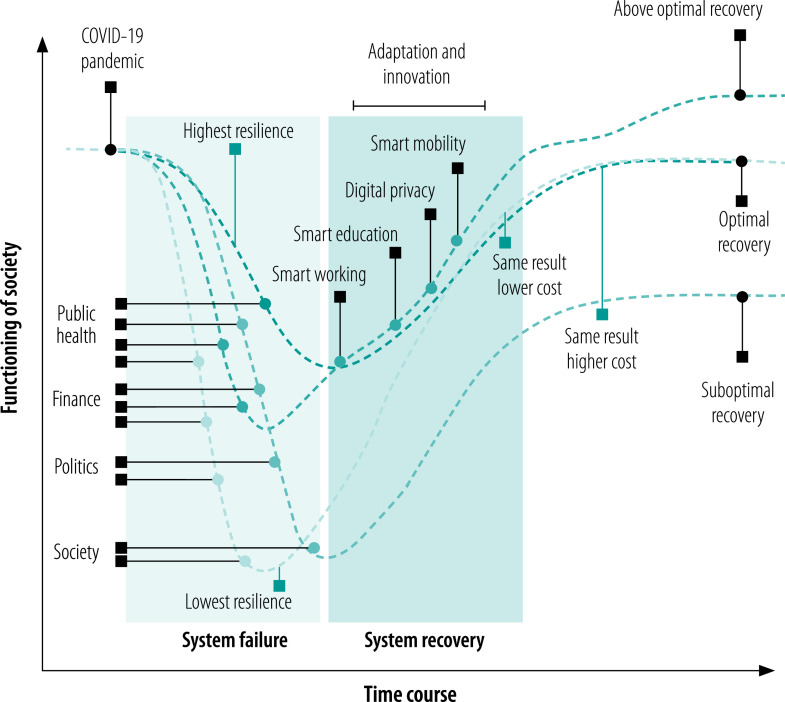
Schematic illustration of plausible scenarios of the effects of shocks due to COVID-19 on society functioning and subsequent recovery

What these examples show is that, after the initial shock and the systemic failures from policy unpreparedness (e.g. in public health, finance, politics and society), there is a crucial phase of adaptation and innovation, the management of which determines the recovery scenario that is likely to emerge. Several scenarios are plausible, depending on initial resilience and, overall, on the ability to adapt to changes (Fig. 3). If a prompt, effective redesign of the whole system of socioeconomic interdependencies has been done and mapped by an appropriate multilayered model, there is an opportunity to capitalize on the disruption of the status quo and put in place structural changes that would not have been feasible through business-as-usual policy negotiation, which is generally much more limited in scope and with a relatively narrow focus. Clearly, the best recovery scenario requires a speedy and targeted response that minimizes damage. Given that socioeconomic inequality has a negative effect on people’s behavioural responses to policy measures, the best scenario will be facilitated by low levels of inequality.^30^ A high level of social coordination and governance is required to enable best-response scenarios. This coordination and governance must be carefully pursued as a precondition for effective public health interventions and not only regarded as an exit strategy from the current crisis; indeed, it is even more important in the post-pandemic context. The less systematic the redesign of the system, then: the slower and more ineffective the response will be; the deeper the socioeconomic inequalities will become; the more the structural adjustment will be limited; and the more the recovery will fall into less and less effective scenarios. We briefly outline three possible recovery scenarios after COVID-19 in Box 1.

COVID-19: coronavirus disease 2019.

Note: The plausible recovery scenarios are explained in Box 1.

Box 1 **Plausible recovery scenarios after COVID-19**

**Scenario 1: suboptimal**

The aftermath. The global economic system fails to coordinate for a successful economic recovery. Long periods of economic stagnation occur with mounting social unrest. Populist political narratives increase locally, threatening the global architecture of free trade and division of labour. Socioeconomic inequalities are exacerbated and concern for environmental challenges is played down.

**Scenario 2: optimal**

The so-called new normal. A substantial economic recovery package is successfully deployed on a global scale and pre-crisis levels of economic activity gradually return. New guidelines for public life in a post-pandemic scenario are developed and successfully implemented. The organization of social and economic life adapts to the new challenges with no major organizational breakdowns.

**Scenario 3: above optimal**

A coordinated, multilayered innovation push. A great wave of social and technological innovation with strong public support starts a new growth cycle. Innovations in working, education and movement redesign the organization of cities, living and working spaces, with a positive impact on climate. Public policies comprehensively target socioeconomic inequalities.

## Integrative approach

From the perspective of systems science, we are probably in the middle of a critical phase of transition, where small differences in choices may accumulate into very different long-term trajectories. Pandemics have often been turning points, for better or for worse. Whereas the end of the Roman Empire was greatly accelerated by the effects of the perhaps first historically recorded pandemic, the so-called plague of Justinian,^31^ historians point out how the Black Death pandemics of the late Middle Ages not only paved the way to the human flourishing of the Renaissance period,^32^ but are at the origin of public health as a scientific discipline.^33^

If policy preparedness is key to resilience against large systemic shocks, how should we move forward now? Over-simplifying the lessons learnt by a straightforward application of linear thinking must be avoided. For instance, while it is still to be understood why the response to the pandemic in European and North American countries was slower than in Asia, one could speculate that the quick response of Asian countries was due to command-and-control mechanisms that are only available to authoritarian governments. The implication would then be that democracies are less capable of implementing the quick targeted policy responses that are needed after a large, unexpected crisis. This assumption would be a naive interpretation built on the characteristics of a single-system layer and linear cause–effect relationships, and the available evidence does not support this opinion.^34^ The responses of Hong Kong Special Administrative Region, Republic of Korea and Singapore were exemplary, and all based on proactive and extensive contact-tracing through technology and mass adoption of protective face masks. These countries have different levels of democratic orientation but a common culture that emphasizes personal responsibility in the public interest.^35^

To arrive at a truly integrative approach to public health in a hyper-connected global socioeconomic environment such as the present one, we need to assign a new, pivotal role to computational social science as an analytical toolbox for data-driven policy design through a self-reinforcing cycle of model making, analysis, simulation and post-validation. Relying on simple explanations and the isolation of a small number of domain-relevant factors while not considering the big picture does not allow an effective response to large systemic shocks such as the COVD-19 pandemic. Furthermore, this limited approach works even less well in imagining innovative recovery strategies that exploit the disruption of the status quo to achieve important sustainability goals, which otherwise may have been unreachable. To achieve an integrative approach requires a new dialogue and alliance between many different fields such as public health, medicine, social sciences, applied physics, economics and possibly more. Computational social science may offer the appropriate transdisciplinary platform to bring about this dialogue to flourish and allow us to tackle, in the public interest, the formidable social challenges ahead of us with a new spirit and renewed energy.
